# Physicochemical Investigations of Homeopathic Preparations: A Systematic Review and Bibliometric Analysis—Part 2

**DOI:** 10.1089/acm.2019.0064

**Published:** 2019-09-12

**Authors:** Alexander Tournier, Sabine D. Klein, Sandra Würtenberger, Ursula Wolf, Stephan Baumgartner

**Affiliations:** ^1^ Institute of Complementary and Integrative Medicine, Faculty of Medicine, University of Bern, Bern, Switzerland.; ^2^ Water Research Lab, Heidelberg, Germany.; ^3^ Scientific and Regulatory Affairs, Hevert-Arzneimittel GmbH & Co. KG, Nussbaum, Germany.; ^4^ Society for Cancer Research, Arlesheim, Switzerland.; ^5^ Institute of Integrative Medicine, Faculty of Health, University of Witten/Herdecke, Witten, Germany.

**Keywords:** physics, very high dilutions, serially diluted and agitated solutions, ultrahigh aqueous dilutions

## Abstract

***Objectives:*** In Part 1 of the review of physicochemical research performed on homeopathic preparations the authors identified relevant publications of sufficient reporting quality for further in-depth analysis. In this article, the authors analyze these publications to identify any empirical evidence for specific physicochemical properties of homeopathic preparations and to identify most promising experimental techniques for future studies.

***Methods:*** After an update of the literature search up to 2018, the authors analyzed all publications in terms of individual experiments. They extracted information regarding methodological criteria such as blinding, randomization, statistics, controls, sample preparation, and replications, as well as regarding experimental design and measurement methods applied. Scores were developed to identify experimental techniques with most reliable outcomes.

***Results:*** The publications analyzed described 203 experiments. Less than 25% used blinding and/or randomization, and about one third used adequate controls to identify specific effects of homeopathic preparations. The most promising techniques used so far are nuclear magnetic resonance (NMR) relaxation, optical spectroscopy, and electrical impedance measurements. In these three areas, several sets of replicated high-quality experiments provide evidence for specific physicochemical properties of homeopathic preparations.

***Conclusions:*** The authors uncovered a number of promising experimental techniques that warrant replication to assess the reported physicochemical properties of homeopathic preparations compared with controls. They further discuss a range of experimental aspects that highlight the many factors that need to be taken into consideration when performing basic research into homeopathic potentization. For future experiments, the authors generally recommend using succussed (vigorously shaken) controls, or comparing different homeopathic preparations with each other to reliably identify any specific physicochemical properties.

## Introduction

Homeopathy is a very popular complementary medicine worldwide.^1^ Scientific interest into therapeutic efficacy of homeopathic remedies is reflected in the growing number of studies and meta-analyses in clinical research.^2^ However, specific efficacy and the mode of action of homeopathic remedies—especially in high dilution—is still the subject of scientific debate.

A major challenge of homeopathic basic research is to decode any physicochemical mode of action. The aim of this review project was to contribute to this effort through a systematic literature search and a thorough evaluation of the state of research in this field.

In Part 1 of this review the authors presented the methodology of the literature search and a bibliometric classification of the publications that were found.^3^ In the present, second article, the authors report on a methodological analysis of the investigations in the different physicochemical research areas. The authors focus on criteria such as blinding, randomization, use of statistics, controls, independent sample preparation, and replicated experiments. The aim was to identify any empirical evidence for specific physicochemical properties of homeopathic preparations, and to identify most promising experimental techniques for future studies. In the forthcoming Part 3 of this review, a qualitative in-depth analysis of the research field will be performed to get clues about possible hypotheses for the physicochemical mode of action.

## Materials and Methods

In Part 1 of this review the authors described how they performed the literature search and the procedure by which they assigned to each publication a Manuscript Information Score (MIS) as a proxy for the reporting quality of the article.^3^ A total of 183 publications on physicochemical experiments in homeopathy were retrieved; 122 of these had an MIS ≥5 and were thus considered to be of sufficient quality to be included in this part of the review. On closer inspection seven of these publications were found not to be relevant to this review and were excluded (six because of duplications^4–9^ and one because it was not standard homeopathy^10^).

The authors performed an update of the literature search to cover all publications up to December 2018. The same search criteria and methods as previously were used.^3^ The update uncovered 19 publications, reporting on 32 experiments. In this part of the review the authors analyzed these 134 publications in terms of the 203 experiments they describe.^9,11–143^ The definition of an experiment was taken as an intellectually conceivable unit with uniform measurement methods and a homogenous set of samples. Thus, the authors split publications into multiple experiments when different measurements procedures and/or differing sample preparation methods had been applied. An experiment could be described in several publications and quite often a publication would contain several relevant experiments.

Detailed information for each experiment was extracted from the publications (see Box 1).

All data were independently extracted by two authors and compared. Conflicts were resolved through discussion. The detailed data extraction sheet is available as Supplementary Data. Two authors worked on each of the 11 research areas. Because of the search criteria excluding nonhomeopathy-specific articles, some work potentially relevant to the mode of action of homeopathic dilutions were not included (see Limitations section).

To quantitatively compare any empirical evidence for specific effects of homeopathic preparations for the 11 research areas, we constructed a methodological score—the Methodological and Frequency of Investigation (MFI) score—by taking the average of 5 quality criteria (randomization, blinding, statistics, use of succussed controls, and use of multiple independent production lots) and weighing it by the number of studies in that research area.

BOX 1 General information

Authors, year of publication, title of publication, research area (one of 11 predefined categories), and type of Publication (journal, book, etc.) were the general information.

Homeopathic preparations

Homeopathic preparations included potentized substances (Latin or chemical names), method of potentization (multiple tube vs. single tube, hand vs. machine, succussion vs. vortex vs. sonication), potency levels and dilution ratio, Substance of potency vessel, composition of potentization medium, production of potencies (self-made, off the shelf, and specific external production), and physical modifications.

Controls

Types of controls (unsuccussed medium, dilution without succussion, succussed medium, potentized medium, none, and other).

Bias prevention and statistics

Number of independent homeopathic and control sample production lots; blinding, randomization, statistics (none, descriptive, inferential).

Techniques and results

Measurement technique in more detail, summary of results, results reported by authors (evidence or no evidence for empirical differences between homeopathic preparations and controls).

## Results

### Formal overview

**FIG. 1.**
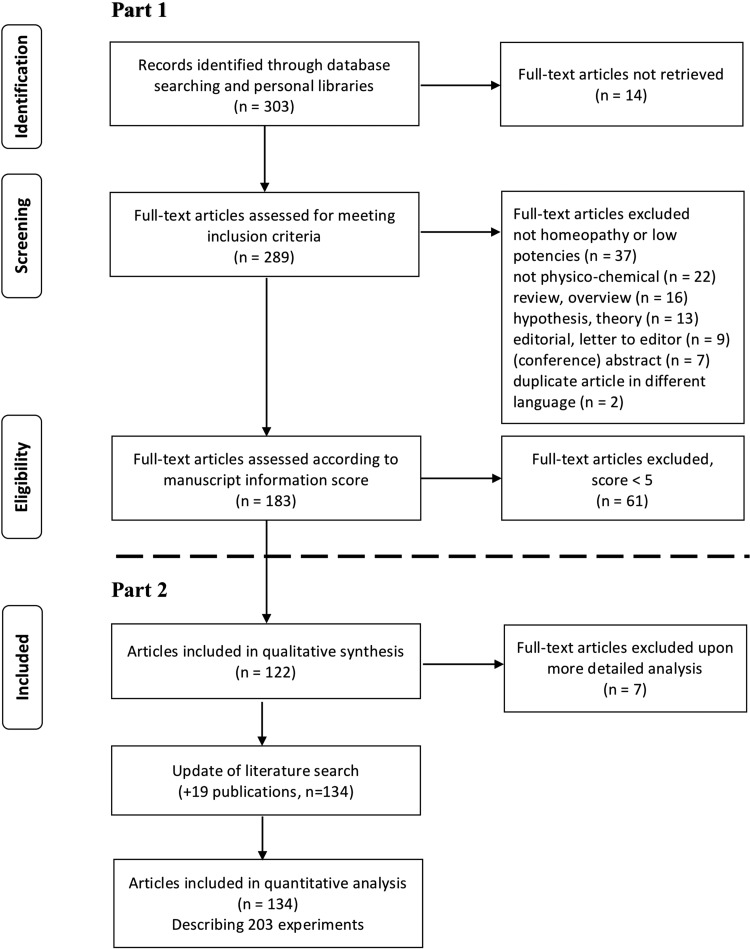
PRISMA flow diagram describing the process of paper inclusion through Part 1 and Part 2 of the review to arrive at the 203 experiments being described. PRISMA, Preferred Reporting Items for Systematic Reviews and Meta-Analyses.

The 134 publications analyzed reported on a total of 203 experiments (Fig. 1). The majority of experiments (72%) reported findings in line with the notion that homeopathic preparations are different from the controls used (Table 1).

**Table 1.** Physicochemical Experiments Reporting Differences Between Homeopathic Preparations and Controls

*Findings*	*Count*	*%*
Differences reported	147	72
No differences reported	35	17
Mixed results	2	1
NA	19	9

Most experiments used plain (unsuccussed) potentizing medium as control, whereas less than one third used potentized medium (Table 2); 23% of experiments used more than one control. Twenty-three percent of experiments reported blinding and 21% reported randomization. Only 15% of the experiments reported measurements of multiple independent sample production lots. Most of the experiments (59%) did not report the use of statistics to analyze the data, whereas 28% reported use of inferential statistics.

**Table 2.** Controls, Bias Prevention and Statistics Used in Physicochemical Experiments

	*Count*	*%*
Controls^a^		
Potentized medium	57	28
Succussed medium	18	9
Dilution without succussion	24	12
Unsuccussed medium	109	54
Other	50	25
Unknown	15	7
Blinding		
Yes	46	23
No	22	11
Not described	135	67
Randomization		
Yes	43	21
No	20	10
Not described	140	69
Independent production lots		
Unique production lot	43	21
Multiple production lots	31	15
Unknown	129	64
Statistics		
Inferential	56	28
Descriptive	27	13
None	120	59

Types of controls used in experiments, and information on whether sample blinding and randomization was used, whether experiments used independent production lots (i.e., how many times the homeopathic preparations were produced from the original substance) and whether statistics were used and what type.

^a^ Experiments often used several controls.

Table 3 reports on specific aspects of the sample production. Two thirds of the experiments used the multiple-tube method for potentization. Nearly half of the experiments declared use of some form of potentizing machine. The majority of experiments used succussion as potentization method. About two thirds of the preparations investigated were prepared by the researchers in their own laboratory. Experiments most often investigated samples both above and below the theoretical limit where nothing would be left of the original substance (12c/24x, roughly corresponding to the inverse of Avogadro's number). Although the assumption is generally that the effects of homeopathy are the result of as yet unknown water characteristic, only 24% of experiments used ultrapure water as the medium for the dilution/succussion process. Most experiments (35%) used ethanol at different concentrations. Of interest, 20% of experiments used specifically prepared water-based mediums such as solutions of sodium bicarbonate, silicic acid, sodium chloride, and lithium chloride.

**Table 3.** Homeopathic Sample Preparation

	*Count*	*%*
Potentizing method		
Multiple tube (Hahnemann)	124	61
Single tube (Korsakov)	11	5
Mixed	25	12
Unknown	43	21
Succussion method		
Hand	56	28
Machine	81	40
Mixed	10	5
Unknown	56	28
Production		
Self-made	119	59
Specific external	40	20
Off the shelf	27	13
Mixed	17	8
Potencies		
<12c, 24x	17	8
≥12c, 24x	42	21
Mixed	141	69
Unknown	3	1
Composition of potentization medium		
Ethanol high concentration (>50%)	56	28
Ethanol low concentration	15	7
Ultrapure water	49	24
Water-based inorganic solutions	40	20
Mixed	13	6
D_2_O	3	1
Unknown	27	13

Methods that were employed in the production of the samples: potentizing method (multiple- or single-vessel method), succussion method (hand, machine), production location (in-house or outsourced), and dilution level (below or above the inverse Avogadro number, i.e., whether any of the original substance could be expected to remain in the sample).

A total of 192 different substances were investigated. The most used potentized substance was *Natrium muriaticum* (sodium chloride), followed by 2.4-dichlorophenoxyacetic acid, *Arnica montana*, sulfur, *Nux vomica*, and *Silicea* (Table 4).

**Table 4.** Substances Potentized Most Used in Experiments

*Remedies*	*Usage*
*Natrium muriaticum*	28
2,4-dichlorophenoxyacetic acid	24
*Arnica montana*	24
Sulfur	21
*Nux vomica*	17
*Silicea*	15
*Argentum nitricum*	12
*Arsenicum album*	12
*Argentum metallicum*	11
*Cuprum sulfuricum*	10
*Magnesium muriaticum*	10
*Arsenicum sulphuratum rubrum*	9
*Plumbum nitricum*	7
*Aurum muriaticum*	7
*Lycopodium clavatum*	6
*Aurum metallicum*	6
*Zincum metallicum*	6
*Histaminum*	5
*Zincum oxidatum*	5
*Alcoholus* (*ethanol*)	5
*Bryonia*	5

Usage in number of experiments.

The most frequently used measurement techniques were electrical impedance, spectroscopy followed by nuclear magnetic resonance (NMR) (Table 5). From the breakdown per method we see that blinding and randomization was used most often in NMR, although still less than half of the experiments used them. Use of inferential statistics was generally low except for NMR where they were used often (77%). In terms of the use of succussed controls, these were used less than half of the time except for NMR (63%). The use of independent production lots was generally very low except for NMR (33%) and chromatography (100%). According to the methodological score applied (MFI score, see Materials and Methods section), NMR comes out as the method with the most reliable empirical evidence for specific properties of homeopathic preparations (MFI score of 15.8), followed by spectroscopy techniques (MFI score of 10.2), analytical methods (6.2), and electrical impedance (MFI score of 6.0).

**Table 5.** Experimental Methods Used

*Methods*	*Count*	*Blinding (%)*	*Randomization (%)*	*Statistics (%)*	*Succussed controls (%)*	*Independent production lots (%)*	*MFI score*	*Differences reported (%)*
NMR	30	40	50	77	63	33	15.8	73
Spectroscopy	39	31	21	31	38	10	10.2	79
Analytical methods^a^	22	27	32	14	45	23	6.2	18
Electrical impedance	41	12	12	12	22	15	6.0	80
Imaging methods	16	25	25	38	50	6	4.6	69
Surface tension/various physical	9	33	22	22	56	11	2.6	56
Luminescence	11	18	18	27	36	9	2.4	100
Calorimetry	16	0	0	13	13	0	0.8	94
Raman spectroscopy	7	29	0	0	29	0	0.8	86
Chromatography	3	0	0	0	0	100	0.6	100
Electrochemistry	9	0	0	0	11	0	0.2	89

Ordered by MFI score (see the Materials and Methods section). Count, number of experiments per method. Blinding, randomization, inferential statistics, succussed controls, and independent production lots, frequency of use. Differences between homeopathic preparations and controls as reported in publication: frequency.

^a^ The low level of reported differences in the Analytical Methods group is because of the fact that many experiments used analytical methods to control sample purity, not as a technique to compare samples.

MFI, Methodological and Frequency of Investigation.

If we look at the best experiments, defined as those that used blinding, randomization, and inferential statistics, we find overall 24 experiments, of which 79% reported differences between homeopathic preparations and controls (Table 6). Of those, 10 fulfilled a further two methodological criteria (use of succussed controls and use of independent lot production), 80% of which reported differences between homeopathic preparations and controls.

**Table 6.** High-Quality Experiments

*Method*	*Experiments fulfilling three criteria*	*Reported differences (%)*	*Experiments fulfilling five criteria*	*Reported differences (%)*
NMR	10	90	6	100
Spectroscopy	7	86	1	100
Imaging methods	4	75	1	0
Analytical methods	2	0	0	—
Surface tension/various physical	2	100	2	50
Electrical impedance	2	50	0	—
Luminescence	2	100	0	—
Grand total	29	79	10	80

Number of experiments fulfilling three quality criteria (blinding, randomization, and inferential statistics) along with percentage of experiments reporting differences, number of experiments fulfilling an additional two quality criteria (succussed controls and independent series production) along with corresponding percentages of experiments reporting differences.

NMR, nuclear magnetic resonance.

### Replications

For the purposes of this review, we define a replication as an experiment that used the same investigative technique to measure the same physicochemical properties of homeopathic potencies made of the same substances. Note that this is different from a reproduction where the same instrument and potencies would have to be used, along with the same statistical analysis, experiment protocol, and materials.

We extracted the replication data for all research techniques. The data tables summarizing these replications for each of the 11 experimental methods are given in the Supplementary Tables S1, S2, S3, S4, S5, S6, S7, S8, S9, S10, S11. A synthesis of the replication data is given in Table 7. To score the different replications, we defined the Experimental Replication (ER) score as the number of replications times the average methodological score of these replications times the percentage of experiments reporting differences. This score enables to determine which replications have most reliably reported differences between homeopathic preparations and controls and should therefore be replicated further to confirm (or not) their results.

**Table 7.** Replicated Experiments

*Technique*	*Replication series*	*No. of replications*	*Average MFI score*	*No. of reported differences (%)*	*ER score*
NMR	T1, T2: *Silicea*	7	4.4	100	31.0
NMR	T1, T2: Histamine	5	3.4	100	17.0
Spectroscopy	UV: Sulfur	4	4.0	100	16.0
Spectroscopy	UV: *Cuprum sulfuricum*	3	4.3	100	13.0
NMR	T1, T2: Sulfur	9	2.1	44	8.4
Electrical impedance	REDEM: *Argentum nitricum*	2	4	100	8.0
Electrical impedance	REDEM: *Aurum*	2	4	100	8.0
NMR	T1, T2: *Nux vomica*	3	2.3	100	7.0
Luminescence	Thermo: *Lithium muriaticum*	3	2.0	100	6.0
Imaging methods	GDV: *Natrium muriaticum*	2	2.5	100	5.0
Spectroscopy	UV: *Aconitum napellus*	3	1.7	100	5.0

Number of replications of a given experiment, showing average methodological (MFI) score of experiments within a replication, how often differences between homeopathic preparations and controls were reported and associated ER score. Replications with ER score ≥5 sorted according to ER score.

ER, Experimental replication; GDV, gas discharge visualization; MFI, Methodological and Frequency of Investigation; NMR, nuclear magnetic resonance; UV, ultraviolet.

We see that the seven replications investigating T1 and T2 NMR relaxation times of potentized silica have high methodological scores and that all seven experiments reported differences between homeopathic preparations and controls, leading to a high ER score of 31.0. Similarly, NMR relaxation time investigations of potentized histamine have a high score of 17.0 with 5/5 replications reporting differences.

In spectroscopy, we have two sets of ultraviolet (UV) measurements (*Cuprum sulfuricum*, sulfur potencies) with high ER scores. In electrical impedance, the two replications of the REDEM experiments (black box measurements) stand out as having high methodological scores, and both replication lines reported differences between homeopathic preparations and controls leading to a decent ER. We also see the three studies of thermoluminescence using homeopathic preparations of *Lithium muriaticum*.

## Discussion

### Appropriate controls for potentized preparations

A major question that came up during this review is how to define the most appropriate controls for homeopathic preparations in physicochemical measurements. We can distinguish two main classes of controls: (1) plain (unsuccussed) solvent or diluted (but not succussed) homeopathic samples and (2) potentized or succussed (vigorously shaken) solvent. It is quite evident that succussion of a fluid in ambient air leads to a number of effects such as formation of air bubbles of different size with differential lifetimes, increased dissolution of air components (N_2_, O_2_, CO_2_) in the fluid, increased dissolution of potentization vessel wall material (Si, B, Na, K etc.), and maybe cavitation effects.^144^ These processes may lead to further consequences such as increased oxidative processes (because of O_2_ dissolution), changes in pH (because of CO_2_ dissolution and acid formation), changes in nuclear magnetic relaxation (O_2_ as relaxation agent), increased silica-hydrogel formation (because of increased Si dissolution), radical formation (because of cavitation), and potentially other effects.

When investigating the hypothesis that potentization of a given material leads to remedies with specific effects, it is evident that any such specific effects have to be different from pure succussion effects that are unspecific in the sense that they are not related to the substance potentized. From this point of view, only succussed or potentized controls are valid controls for demonstrating any evidence for specific (remedy-related) properties of potentized preparations.

Considering the hypothesis that succussion leads to some information transfer of the substance potentized to the potentization medium, the question arises what happens when pure medium is potentized as control sample (e.g., potentized water). One could speculate that the potentization process is amplifying some random information. This would lead to a situation where samples with specific information (homeopathic preparations) would be compared with samples with random information (potentized water as control). In this sense potentized medium (and by extension succussed medium too) might not to be the best controls possible as they could introduce a random element.

On the contrary, one can argue that it would not be wise to compare homeopathic preparations with each other in case the measurement method used is not able to distinguish the putative homeopathic structures. Because the nature of the homeopathic structures is not known yet, it cannot be decided at present if a given measurement method is able or not to distinguish the presumed structures.

We therefore recommend in future investigations the use of two types of controls: (1) potentized solvent and (2) other homeopathic preparation(s). The use of several homeopathic samples increases the probability to identify different structures. Furthermore, if possible, we recommend the additional use of (3) unsuccussed and (4) succussed control samples that would allow determining the effect of pure succussion. A study of the effect of succussion on the observed physicochemical activities would be a welcome endeavor as it is currently lacking, in particular investigating the effect of the number of succussions would be very interesting. Such a study would complement the work by Betti et al., which used wheat germination assays and the droplet evaporation method to explore this topic, showing a sigmoidal type behavior as a function of the number of succussions.^145^

As mentioned previously, certain experimental methods might be more suitable to investigating the presence/absence of such structures rather than distinguishing between such structures. The issue here is in conceiving appropriate controls that would not suffer from confounding factors such as gas dissolution for example. Such an approach is being pioneered by the group of Prof. Elia in their conductivity measurement experiments; they used trace analytics methods to levels of sodium in their samples and are thereby able to calculate the theoretical conductivity of the sample (sodium being the most relevant element for conductivity in their setup) to obtain the so-called “excess conductivity” as the difference between the measured conductivity and the theoretical prediction. This technique is interesting as in principle it probes directly for the presence of structures with unexplained properties (here in terms of conductivity). However, the technique does not currently control for other elements and gases originating from the succussion process that could also play a role.

### Bias prevention and statistics

Most of the studies neither used blinding nor randomization. This is not entirely unusual in physicochemical research where one usually does not expect experimenter effects. Most of conventional research does not invoke blinding on such experiments for that very reason. Giving the history and heated debate surrounding homeopathy, we recommend implementing blinding and randomization protocols in future investigations to ensure that experimenters do not have any effect on the results.

Rather worrying is the lack of use of proper statistical tools. Part of the problem here is that many of the studies are quite dated and statistical tools were often not used at that time. Another effect is that many experiments such as in spectroscopy have been rather descriptive and therefore did not use statistical tools. Now, again given the controversy in the field, there is a great need for proper statistical methods to be implemented, so as to quantify the degree of uncertainty in the results and to avoid Type I and Type II errors.

We recommend the implementation of systematic negative control (SNC) experiments on a regular basis. SNC experiments are full experiments with identical design and evaluation as experiments with homeopathic preparations, but all samples are either from the same source material (e.g., plain potentization medium) or consist of potentized medium, prepared analogously as the homeopathic samples.^146^ Depending on the design of the experiments, systematic positive control (SPC) experiments may also be a valid approach, consisting of the same sample (either a positive control or a homeopathic preparation), independently prepared in the number of samples assessed in the “true” experiments. SNC and SPC experiments are excellent scientific tools to evaluate the stability of a given experimental system, to identify any systematic error, and to assess applicability of statistical models. In this review, only one investigation implemented SNC experiments.^97^

Another aspect that needs to be addressed in the future is the inherent variability present in physicochemical studies of homeopathic remedies. It is quite clear that there is a high degree of variability in the experimental measurements,^139^ which cannot be solely attributed to instrumental error and are because of the high variability inherent in water itself.^147^ SNC and SPC experiments are well suited to address these issues. In addition, adapted statistical models may be necessary to address variability in itself.

### Most promising techniques

Looking at the results gathered in this review a number of experiments emerge as deserving further replication and exploration. First of all, NMR relaxation studies of potentized silica and histamine preparations have shown the most methodological rigor and the most promising results, demonstrating the ability to distinguish between potentized silica or histamine, and potentized controls. Of interest, potentized sulfur seems to be harder to distinguish from corresponding controls.

Based on the available data, UV spectroscopy seems to be the second most interesting technique. With this experimental approach only, a formal meta-analysis over three independent experimental series yielded statistically significant differences between potencies of copper sulfate and succussed medium.

Thermoluminescence on potentized lithium chloride seems to be the third promising technique, although it requires quite sophisticated and expensive equipment.

In contrast, both NMR relaxation and UV spectroscopy can be performed with desktop instruments, and can additionally be equipped with autosamplers to allow a high number of samples to be measured. From a pragmatic point of view, these two methods therefore seem to be most promising to be recommended for further replication studies.

Of interest, two unconventional experimental methods (so-called REDEM spectroscopy and gas discharge visualization) also seem to have a potential to distinguish between potentized preparations (silver nitrate, gold, sodium chloride) and controls. The disadvantage of these approaches is that the exact measurement process is only partially understood, and cannot be scientifically interpreted in a straightforward way.

### Limitations

The search criteria defined homeopathic preparations as having undergone succussion steps, as such a number of publications from the field of water research and of high-dilutions research were not included. In particular the work of Pollack on “Exclusion Zone” (EZ) water, which is often cited as a possible line of enquiry for explaining homeopathy, did not fulfill the criteria and was not retained (for a overview of this field, the reader is referred to the book by Pollack: “The Fourth Phase of Water”^148^). Similarly, the work of Konovalov and Ryzhkina on structures terms “Nanoassociates” at ultralow dilutions, did not meet the criteria and was not retained (for more details the reader is referred to the review of the field by Konovalov and Ryzhkina^149^). It is quite clear that the homeopathic remedy production process with iterative dilution and succussion steps raises fundamental questions in the realm of physics and chemistry that go far beyond the field of homeopathy research. A review on water structures and water/ethanol structures and on physicochemical effects of succussion would be a very valuable complementary approach for homeopathic basic research.

## Conclusions

We reviewed 134 publications describing 203 experiments in the area of physicochemical research into homeopathically potentized preparations, which we analyzed in detail with the aim of extracting relevant information about what has been learned in the field and which experiments to undertake in the future.

To conclude, the most promising techniques used so far are NMR relaxation, optical spectroscopy, and electrical impedance measurements. In these three areas, several sets of replicated high-quality experiments provide evidence for specific physicochemical properties of homeopathic preparations.

For future experiments, we recommend using succussed controls, or comparing different homeopathic preparations with each other to reliably identify any specific physicochemical properties. We also recommend the use of systematic positive and negative control experiments as a way of measuring the inherent variability in an experimental setup.

Further in-depth analysis of the experiments published is warranted to extract hypotheses regarding a possible mode of action of potentized remedies; such an analysis will be published as Part 3 of this review.

## Supplementary Material

Supplemental data

Supplemental data

Supplemental data

Supplemental data

Supplemental data

Supplemental data

Supplemental data

Supplemental data

Supplemental data

Supplemental data

Supplemental data

Supplemental data

## References

[1] Bodeker G, Ong C-K, Grundy C, et al. WHO Global Atlas of Traditional, Complementary and Alternative Medicine. Kobe: WHO Centre for Health Development, 2005.

[2] Mathie RT, Lloyd SM, Legg LA, et al. Randomised placebo-controlled trials of individualised homeopathic treatment: Systematic review and meta-analysis. Syst Rev 2014;3:142 25480654 10.1186/2046-4053-3-142 PMC4326322

[3] Klein SD, Wurtenberger S, Wolf U, et al. Physicochemical investigations of homeopathic preparations: A systematic review and bibliometric analysis—Part 1. J Altern Complement Med 2018;24:409–421 29377709 10.1089/acm.2017.0249 PMC5961874

[4] Mayrhofer C. Microscopic investigations of homeopathic metal preparations [In German]. Hygea 1842;16:97–106

[5] Witt C. Physical investigation of high homeopathic potencies [In German]. Essen: KVC Verlag, 2000.

[6] Roder E, Frisse R On the stability of homeopathic dilutions in glass and plastic containers. Pharmazie 1981;36:615–619 7301902

[7] Demangeat JL, Gries P, Poitevin B Modification of 4 MHz NMR water proton relaxation times in highly diluted aqueous solutions. Br Homeopath J 1995;84:169–170

[8] Weingärtner O. NMR-features that relate to homoeopathic sulphur-potencies. Berlin J Res Homoeoepathy 1990;1:61–68

[9] Anagnostatos GS, Pissis P, Viras K, Soutzidou M Theory and experiments on high dilutions. In: Ernst E, Hahn EG, eds. Homoeopathy—A Critical Appraisal. Oxford: Butterworth-Heinemann, 1998:153–166

[10] Resch G, Gutmann V, Schauer H The “shaking effect” on the conductivities of liquids. J Ind Chem Soc 1982;59:130–132

[11] Aabel S, Fossheim S, Rise F Nuclear magnetic resonance (NMR) studies of homeopathic solutions. Br Homeopath J 2001;90:14–20 11212083 10.1054/homp.1999.0458

[12] Anick DJ. High sensitivity ^1^H-NMR spectroscopy of homeopathic remedies made in water. BMC Complement Altern Med 2004;4:15 15518588 10.1186/1472-6882-4-15 PMC534805

[13] Assumpcao R. Electrical impedance and HV plasma images of high dilutions of sodium chloride. Homeopathy 2008;97:129–133 18657771 10.1016/j.homp.2008.06.003

[14] Bandyopadhyay P, Basu R, Das S, et al. Enhancement of quantum efficiency of a dye-sensitized electrochemical cell by using triturated zinc oxide mixed with two organic dyes, Azure C and Rose Bengal. Int J High Dil Res 2017;16:1–6

[15] Baumgartner S, Wolf M, Skrabal P, et al. High-field ^1^H T_1_ and T_2_ NMR relaxation time measurements of H_2_O in homeopathic preparations of quartz, sulfur, and copper sulfate. Naturwissenschaften 2009;96:1079–1089 19533076 10.1007/s00114-009-0569-y

[16] Beier K. On physical effects or properties of true high homeopathic potencies [In German]. [Thesis]

[17] Bell IR, Lewis DA, Brooks AJ, et al. Gas discharge visualization evaluation of ultramolecular doses of homeopathic medicines under blinded, controlled conditions. J Altern Complement Med 2003;9:25–38 12676033 10.1089/107555303321222928

[18] Bell IR, Muralidharan S, Schwartz GE Nanoparticle characterization of traditional homeopathically-manufactured *Gelsemium sempervirens* medicines and placebo controls. Nanomed Biotherapeutic Discov 2015;5:136

[19] Bell IR, Muralidharan S, Schwartz GE Nanoparticle characterization of traditional homeopathically-manufactured silver (Argentum Metallicum) medicines and placebo controls. Nanomed Nanotechnol 2015;6:311–316

[20] Belon P, Elia V, Elia L, et al. Conductometric and calorimetric studies of the serially diluted and agitated solutions—On the combined anomalous effect of time and volume parameters. J Therm Anal Calorim 2008;93:459–469

[21] Betti L, Elia V, Napoli E, et al. Biological effects and physico-chemical properties of extremely diluted aqueous solutions as a function of aging-time. Front Life Sci 2011;5:117–126

[22] Bhattacharyya SS, Mandal SK, Biswas R, et al. In vitro studies demonstrate anticancer activity of an alkaloid of the plant *Gelsemium sempervirens*. Exp Biol Med 2008;233:1591–1601 10.3181/0805-RM-181 18997108

[23] Bonet-Maury P, Deysine A, Vogeli L-M Investigation of homeopathic dilutions by radioisotopes [In French]. Ann Pharm Franc 1954;12:654–663 14350419

[24] Botha I, Ross AHA A nuclear magnetic resonance spectroscopy comparison of 3C trituration derived and 4C trituration derived remedies. Homeopathy 2008;97:196–201 19371568 10.1016/j.homp.2008.08.008

[25] Boyd WE. Research on the Low Potencies of Homoeopathy, an Account of Some Physical Properties Indicating Activity. London: Heinemann, 1936.

[26] Brucato A, Stephenson J Dielectric strength testing of homeopathic dilutions of HgCl2. J Am Inst Homeopathy 1966;59:281–286 6013466

[27] Cacace CM, Elia L, Elia V, et al. Conductometric and pHmetric titrations of Extremely Diluted Solutions using HCl solutions as titrant A molecular model. J Mol Liq 2009;146:122–126

[28] Cartwright SJ. Solvatochromic dyes detect the presence of homeopathic potencies. Homeopathy 2016;105:55–65 26827998 10.1016/j.homp.2015.08.002

[29] Cartwright SJ. Interaction of homeopathic potencies with the water soluble solvatochromic dye bis-dimethylaminofuchsone. Part 1: pH studies. Homeopathy 2017;106:37–46 28325223 10.1016/j.homp.2017.01.001

[30] Cartwright SJ. Degree of response to homeopathic potencies correlates with dipole moment size in molecular detectors. Implications for understanding the fundamental nature of serially diluted and succussed solutions. Homeopathy 2018;107:19–31 29528475 10.1055/s-0037-1617448 PMC6193369

[31] Chakraborty I, Datta S, Sukul A, et al. Variation in free and bound water molecules in different homeopathic potencies as revealed by their Fourier Transform Infrared Spectroscopy (FTIR). Int J High Dilut Res 2014;13:189–196

[32] Chatterjee A, Paul BK, Kar S, et al. Effect of ultrahigh diluted homeopathic medicines on the electrical properties of PVDF-HFP. Int J High Dilut Res 2016;15:10–17

[33] Chibici-Revneanu C. UV spectroscopic and dielectric measurements of water and highly diluted homeopathic drug solutions [In German]. [Thesis]. Leipzig: Universität Leipzig, Fakultät f. Biowissenschaften, Pharmazie und Psychologie (Institut für Pharmazie), 2005.

[34] Chikramane PS, Kalita D, Suresh AK, et al. Why extreme dilutions reach non-zero asymptotes: A nanoparticulate hypothesis based on froth flotation. Langmuir 2012;28:15864–15875 23083226 10.1021/la303477s

[35] Chikramane PS, Suresh AK, Bellare JR, Kane SG Extreme homeopathic dilutions retain starting materials: A nanoparticulate perspective. Homeopathy 2010;99:231–242 20970092 10.1016/j.homp.2010.05.006

[36] Ciavatta L, Elia V, Napoli E, Niccoli M New physico-chemical properties of extremely diluted solutions. Electromotive force measurements of galvanic cells sensible to the activity of NaCl at 25 degrees C. J Solution Chem 2008;37:1037–1049

[37] de Alvarenga ES, de Oliveira APM, da Silva RTB, Casali VWD Effect of magnesium phosphoricum 12c on sodium dodecylsulphate by 13C nuclear magnetic resonance. Int J High Dil Res 2009;8:3–8

[38] Demangeat J-L. NMR water proton relaxation in unheated and heated ultrahigh aqueous dilutions of histamine: Evidence for an air-dependent supramolecular organization of water. J Mol Liq 2009;144:32–39

[39] Demangeat J-L. NMR relaxation evidence for solute-induced nanosized superstructures in ultramolecular aqueous dilutions of silica-lactose. J Mol Liq 2010;155:71–79

[40] Demangeat J-L. Nanosized solvent superstructures in ultramolecular aqueous dilutions: Twenty years' research using water proton NMR relaxation. Homeopathy 2013;102:87–105 23622259 10.1016/j.homp.2013.01.001

[41] Demangeat JL. Gas nanobubbles and aqueous nanostructures: The crucial role of dynamization. Homeopathy 2015;104:101–115 25869975 10.1016/j.homp.2015.02.001

[42] Demangeat JL, Demangeat C, Gries P, et al. Modifications of NMR relaxation times at 4 MHz of protons of the solvent in very high saline dilutions of silica/lactose [In French]. Journal de médecine nucléaire et biophysique 1992;16:135–145

[43] Demangeat JL, Gries P, Poitevin B Modification of 4 MHz N.M.R. water proton relaxation times in very high diluted aqueous solutions. In: Bastide M, ed. Signals and Images. Dordrecht: Kluwer Academic, 1997:95–110

[44] Demangeat JL, Gries P, Poitevin B, et al. Low-field NMR water proton longitudinal relaxation in ultrahighly diluted aqueous solutions of silica-lactose prepared in glass material for pharmaceutical use. Appl Magn Reson 2004;26:465–481

[45] Dragan G. Some consideration of coherency in topoenergetic terms, I. high-resolution mixing calorimetry (HRMC) experiments on aqueous solutions. J Therm Anal 1992;38:1497–1508

[46] Elia V, Ausanio G, Gentile FS, et al. Experimental evidence of stable water nanostructures in extremely dilute solutions, at standard pressure and temperature. Homeopathy 2014;103:44–50 24439454 10.1016/j.homp.2013.08.004

[47] Elia V, Baiano S, Duro I, et al. Permanent physico-chemical properties of extremely diluted aqueous solutions of homeopathic medicines. Homeopathy 2004;93:144–150 15287434 10.1016/j.homp.2004.04.004

[48] Elia V, Elia L, Cacace P, et al. “Extremely diluted solutions” as multi-variable systems—A study of calorimetric and conductometric behaviour as a function of the parameter time. J Therm Anal Calorim 2006;84:317–323

[49] Elia V, Elia L, Marchese M, et al. Interaction of “extremely diluted solutions” with aqueous solutions of hydrochloric acid and sodium hydroxide—A calorimetric study at 298 K. J Mol Liq 2007;130:15–20

[50] Elia V, Elia L, Marchettini N, et al. Physico-chemical properties of aqueous extremely diluted solutions in relation to ageing. J Therm Anal Calorim 2008;93:1003–1011

[51] Elia V, Elia L, Montanino M, et al. Conductometric studies of the serially diluted and agitated solutions on an anomalous effect that depends on the dilution process. J Mol Liq 2007;135:158–165

[52] Elia V, Elia L, Napoli E, Niccoli M Conductometric and calorimetric studies of serially diluted and agitated solutions: The dependence of intensive parameters on volume. Int J Ecodyn 2006;1:361–372

[53] Elia V, Marchese M, Montanino M, et al. Hydrohysteretic phenomena of “extremely diluted solutions” induced by mechanical treatments: A calorimetric and conductometric study at 25 degrees C. J Solution Chem 2005;34:947–960

[54] Elia V, Marchettini N, Napoli E, Niccoli M The role of ethanol in extremely diluted solutions. J Therm Anal Calorim 2014;116:477–483

[55] Elia V, Marrari LA, Napoli E Aqueous nanostructures in water induced by electromagnetic fields emitted by EDS: A conductometric study of fullerene and carbon nanotube EDS. J Therm Anal Calorim 2012;107:843–851

[56] Elia V, Napoli E Dissipative structures in extremely diluted solutions of homeopathic medicines: A molecular model based on physico-chemical and gravimetric evidences. Int J Des Nat Ecodyn 2010;5:39–48

[57] Elia V, Napoli E, Niccoli M On the stability of extremely diluted aqueous solutions at high ionic strength—A calorimetric study at 298 K. J Therm Anal Calorim 2008;92:643–648

[58] Elia V, Napoli E, Niccoli M A molecular model of interaction between extremely diluted solutions and NaOH solutions used as titrant. Conductometric and pH metric titrations. J Mol Liq 2009;148:45–50

[59] Elia V, Napoli E, Niccoli M Thermodynamic parameters for the binding process of the OH- ion with the dissipative structures. Calorimetric and conductometric titrations. J Therm Anal Calorim 2010;102:1111–1118

[60] Elia V, Napoli E, Niccoli M On the stability of extremely diluted solutions to temperature. J Therm Anal Calorim 2013;113:963–970

[61] Elia V, Napoli E, Niccoli M, et al. New physico-chemical properties of extremely dilute solutions. A conductivity study at 25 degrees C in relation to ageing. J Solution Chem 2008;37:85–96

[62] Elia V, Napoli E, Niccoli M, et al. New physico-chemical properties of extremely diluted aqueous solutions—A calorimetric and conductivity study at 25 degrees C. J Therm Anal Calorim 2004;78:331–342

[63] Elia V, Niccoli M Thermodynamics of extremely diluted aqueous solutions. Ann N Y Acad Sci 1999;827:241–248 10.1111/j.1749-6632.1999.tb10426.x 10415834

[64] Elia V, Niccoli M New physico-chemical properties of water induced by mechanical treatments. A calorimetric study at 25°C. J Therm Anal Calorim 2000;61:527–537

[65] Elia V, Niccoli M New physico-chemical properties of extremely diluted aqueous solutions. J Therm Anal Calorim 2004;75:815–836 10.1016/j.homp.2004.04.004 15287434

[66] Frisse R. Investigation of adsorption and analysis by neutron activation of homeopathic dilutions [In German]. [Thesis]. Bonn: Hohe Mathematisch-Naturwissenschaftliche Fakultät, Rheinische Friedrich-Wilhelms-Universität, 1981

[67] Gautam RS, Tewari KP, Roper NK, Mishra RK Spectrophotometric Analysis of Potentisation of Euphrasia Officinalis. Hahnemann Glean 1977;44:1–5

[68] Gay A. Presence of a physical factor in homeopathic dilutions [In French]. Lyon: Editions des Laboratoires P.H.R., 1951

[69] Gayen A, Mondal D, Bandyopadhyay P, et al. Effect of homeopathic dilutions of cuprum arsenicosum on the electrical properties of poly(vinylidene fluoride-co-hexafluoropropylene). Homeopathy 2018;107:130–136 29767830 10.1055/s-0038-1626733

[70] Gebhardt A. FTIR-spectroscopic investigations of aqueous and ethanolic homeopathic drugs [In German]. [Thesis]. Leipzig: Fakultät für Biowissenschaften, Pharmazie und Psychologie, 2002.

[71] Güldenstern W. Measurements of fluorescence of the preparation Aesculus Cortex and of the drug Aesculinum [In German]. Der Merkurstab 2001;54:307–312

[72] Heintz E. Physical effects of highly diluted potentised substances [In German]. Die Naturwissenschaften 1941;48:713–725

[73] Heintz E. Remarks to my article: Physical effects of highly diluted potentised substances [In German]. Die Naturwiss enschaften 1942;30:642

[74] Heintz E. A new arrangement to measure physicochemical effects of potencies: the D-probe [In German]. Elemente der Naturwissenschaft 1971;15:33–44

[75] Holandino C, Harduim R, da Veiga VF, et al. Modeling physical-chemical properties of high dilutions, an electrical conductivity study. Int J High Dil Res 2008;7:165–173

[76] Holandino C, Leal FD, de Olivereira Barcellos B, et al. Chapter 3: Mechanical versus handmade succussions, A physical chemistry comparison. In: Bonamin LV, ed. Signals and Images. Contributions and Contradictions About High Dilution Research. New York: Springer, 2008:37–48

[77] Holandino C, Oliveira AP, Homsani F, et al. Structural and thermal analyses of zinc and lactose in homeopathic triturated systems. Homeopathy 2017;106:160–170 28844289 10.1016/j.homp.2017.06.003

[78] Jerman I, Berden M, Škarja M Instrumental measurements of different homeopathic dilutions of potassium iodide in water. Acupunct Electrother Res 1999;24:29–44 10472820 10.3727/036012999816356462

[79] Kar S, Chakraborty M, Nandy P, et al. Characterization and haemocompatibility of Aurum metallicum for its potential therapeutic application. Ind J Res Hom 2017;11:41–47

[80] Klein SD, Sandig A, Baumgartner S, Wolf U Differences in median ultraviolet light transmissions of serial homeopathic dilutions of copper sulfate, *Hypericum perforatum*, and sulfur. Evid Based Complement Alternat Med 2013;2013 10.1155/2013/370609 PMC3562573 23401712

[81] Klein SD, Wolf U Investigating homeopathic verum and placebo globules with UV spectroscopy. Forsch Komplementarmed 2013;20:295–297 10.1159/000354408 24030453

[82] Klein SD, Wolf U Comparison of homeopathic globules prepared from high and ultra-high dilutions of various starting materials by ultraviolet light spectroscopy. Complement Ther Med 2016;24:111–117 26860812 10.1016/j.ctim.2015.12.017

[83] Knauer H. Proof of effects of potentised solutions by physicochemical means [In German]. Acta Homoeopath 1969;13:157–164

[84] Knauer H. Contributions to potency research [In German]. Pforzheim, 1970

[85] Kolisko L. Physiological and physical proof of effectiveness of smallest entities (1923–1959) [In German]. Stuttgart: Arbeitsgemeinschaft anthroposophischer Ärzte, 1959.

[86] Konar A, Sarkar T, Chakraborty I, et al. Raman spectroscopy reveals variation in free OH groups and hydrogen bond strength in ultrahigh dilutions. Int J High Dil Res 2016;15:2–9

[87] Konar A, Sarkar T, Sukul NC, Sukul A Drugs in ultra-high dilution induce changes in the enthalpy associated with loss of crystallization water in lactose. Int J High Dil Res 2018;17:13

[88] Lasne Y. Properties of “homeopathic” solutions, measurement of magnetic relaxation T2 [In French]. [Thesis]. Lyon: Université Claude Bernard Lyon 1, U.E.R. Faculté de Pharmacie, 1986.

[89] Lasne Y, Duplan JC, Mallet JJ Proof of physical signals of diluted dynamized or “homeopathic” solutions [In French]. Bull M.T.S. 2 1985

[90] Lefebvre N, Aubin M, Ferret-Bouin Y, Vrignaud C Etude de dilutions homéopathiques hahnémanniennes à l'aide du glucose marqué au carbone 14. Ann Hom Franc 1978;20:227–235

[91] Lenger K. Homeopathic potencies identified by a new magnetic resonance method: Homeopathy–an energetic Medicine. Subtle Energ Energy Med 2004;15:225–243

[92] Lenger K, Bajpai RP, Drexel M Delayed luminescence of high homeopathic potencies on sugar globuli. Homeopathy 2008;97:134–140 18657772 10.1016/j.homp.2008.05.003

[93] Lenger K, Bajpai RP, Spielmann M Identification of unknown homeopathic remedies by delayed luminescence. Cell Biochem Biophys 2014;68:321–334 23872840 10.1007/s12013-013-9712-7

[94] Lobyshev VI, Tomkevich MS, Petrushanko IY Experimental study of potentiated aqueous solutions. Biophysics 2005;50:416–420 15977836

[95] Lobyshev VI, Tomkevitch MS Luminescence study of homeopathic remedies. In: Priezzhev AV, Coté GL, eds. Optical Diagnostics and Sensing of Biological Fluids and Glucose and Cholesterol Monitoring. Vol. 4263 Moscow: SPIE, 2001:59–64

[96] Luu-d-Vinh C. Homeopathic dilutions, control and study using Raman spectroscopy [In French]. Montpellier: Université de Montpellier, Faculté de Pharmacie et Institut Européen des Sciences Pharmaceutiques Industrielles, 1974.

[97] Maag GW. Investigation of potencies 1x–12x and 1x–33x of silver nitrate [In German]. Dt Z Hom 1932;49:277–285

[98] Maag GW. Investigation of the succession duration of metal potencies [In German]. Dt Z Hom 1933;49:281–286

[99] Mahata CR. Dielectric dispersion studies of some potentised homeopathic medicines reveal structured vehicle. Homeopathy 2013;102:262–267 24050772 10.1016/j.homp.2013.07.003

[100] Marschollek B, Nelle M, Wolf M, et al. Effects of exposure to physical factors on homeopathic preparations as determined by ultraviolet light spectroscopy. ScientificWorldJournal 2010;10:49–61 20062950 10.1100/tsw.2010.15 PMC5763671

[101] Mayrhofer C. Microscopic investigations of homeopathic metal preparations. Illustrated by drawings [In German]. Hygea 1842;16:17–35

[102] Milgrom LR, King KR, Lee J, Pinkus AS On the investigation of homeopathic potencies using low resolution NMR T2 relaxation times: An experimental and critical survey of the work of Roland Conte et al. Br Homeopath J 2001;90:5–13 11212090 10.1054/homp.1999.0457

[103] Nain AK, Droliya P, Manchanda RK, et al. Physicochemical studies of extremely diluted solutions (homoeopathic formulations) of sulphur in ethanol by using volumetric, acoustic, viscometric and refractive index measurements at different temperatures. J Mol Liq 2015;211:1082–1094

[104] Nain AK, Droliya P, Manchanda RK, et al. Physicochemical studies of homoeopathic formulations (extremely diluted solutions) of acidum salicylicum in ethanol by using volumetric, acoustic, viscometric and refractive index measurements at 298.15, 308.15, 318.15 K. J Mol Liq 2016;215:680–690

[105] Paul BK, Kar S, Bandyopadhyay P, et al. Significant enhancement of dielectric and conducting properties of electroactive polymer polyvinylidene fluoride films. An innovative use of *Ferrum metallicum* at different concentrations. Indian J Res Homoeopathy 2016;10:52–58

[106] Pillai MG, Kumar A, Sharma R, Bhasin N LC-MS based workflows for qualitative and quantitative analysis for homeopathic preparation of hydrastis canadensis. Chromatographia 2014;77:119–131

[107] Piñeros LG, Pombo LM, Delgado C, et al. Effects of additional agitation process on the spectrophotometric profiles of homeopathic high dilutions. Int J High Dilut Res 2016;15:10–21

[108] Ramos de Miranda A Water and ultra high dilutions Characterization and phenomenology. In: GIRI, ed. 22nd GIRI Meeting, May 20, 2008, Monte Carlo. Monte Carlo: Eigenverlag, 2008:3

[109] Rao ML, Roy R, Bell IR, Hoover R The defining role of structure (including epitaxy) in the plausibility of homeopathy. Homeopathy 2007;96:175–182 17678814 10.1016/j.homp.2007.03.009

[110] Rey L. Thermoluminescence of ultra-high dilutions of lithium chloride and sodium chloride. Physica A 2003;323:67–74

[111] Rey L. Can low-temperature thermoluminescence cast light on the nature of ultra-high dilutions? Homeopathy 2007;96:170–174 17678813 10.1016/j.homp.2007.05.004

[112] Sacks AD. Nuclear magnetic resonance spectroscopy of homeopathic remedies. J Holist Med 1983;5:172–177

[113] Sarkar T, Konar A, Sukul NC, et al. Raman spectroscopy shows difference in drugs at ultrahigh dilution prepared with stepwise mechanical agitation. Int J High Dil Res 2016;15:2–9

[114] Sarkar T, Konar A, Sukul NC, et al. Vibrational and Raman spectroscopy provide further evidence in support of free OH groups and hydrogen bond strength underlying difference in two more drugs at ultrahigh dilutions. Int J High Dil Res 2016;15:2–10

[115] Sarkar T, Konar A, Sukul NC, et al. Free water molecules and hydrogen bonding form the basis of variation in homeopathic potencies as revealed by vibrational spectroscopy. Int J High Dilut Res 2015;14:8–15

[116] Sharma A, Purkait B Identification of medicinally active ingredient in ultradiluted *Digitalis purpurea*: Fluorescence spectroscopic and cyclic-voltammetric study. J Anal Methods Chem 2012;2012:109058 22606641 10.1155/2012/109058 PMC3347722

[117] Silvio M, Arnaldo P Ultrasonic study of homoeopathic solutions. Br Hom J 1990;79:212–216

[118] Smith RB, Boericke GW Changes caused by succussion on N.M.R. patterns and bioassay of bradykinin triacetate (BKTA) succussions and dilutions. J Am Inst Homeopathy 1968;61:197–212

[119] Sukul A, Sarkar P, Sinhababu SP, Sukul NC Altered solution structure of alcoholic medium of potentized Nux vomica underlies its antialcoholic effect. Br Homeopath J 2000;89:73–77 10826446 10.1054/homp.1999.0365

[120] Sukul NC, Datta S, Sinhababu SP Conformational changes of bovine serum albumin in 4M urea and ultra high dilutions of different drugs. Sci Cult 2007;73:173–175

[121] Sukul NC, De A, Dutta R, et al. Nux vomica 30 prepared with and without succussion shows antialcoholic effect on toads and distinctive molecular association. Br Homeopath J 2001;90:79–85 11341461 10.1054/homp.1999.0470

[122] Sukul NC, Ghosh S, Sinha Babu SP, Sukul A *Strychnos nux-vomica* extract and its ultra-high dilution reduce voluntary ethanol intake in rats. J Altern Complement Med 2001;7:187–193 11327524 10.1089/107555301750164280

[123] Sukul NC, Ghosh S, Sukul A, Sinhababu SP Variation in Fourier Transform Infrared spectra of some homeopathic potencies and their diluent media. J Altern Complement Med 2005;11:807–812 16296914 10.1089/acm.2005.11.807

[124] Sukul NC, Sinhababu SP, Datta SC, et al. Nematotoxic effect of Acacia auriculiformis and Artemisia nilagerica against root-knot nematodes. Allelopathy J 2001;8:65–72

[125] Süss WG Structure and dynamics of high homeopathic potencies—resonance/damping/dedamping measurements (REDEM) [In German]. In: Süss WG, ed. Homöopathische Arzneimittel–wissenschaftliche Grundlagen für die Herstellung, Qualität und Anwendung. Stuttgart: Deutscher Apotheker Verlag, 2004:49–63

[126] Taufiq Khan M Physical aspects related to the problems in potentised drugs. In: Seitschek R, ed. XXVIII. Internationaler Kongress für homöopathische Medizin. Wien: Österreichische Gesellschaft für Homöopathische Medizin, 1973:473–479

[127] Temgire MK, Suresh AK, Kane SG, Bellare JR Establishing the interfacial nano-structure and elemental composition of homeopathic medicines based on inorganic salts. A scientific approach. Homeopathy 2016;105:160–172 27211323 10.1016/j.homp.2015.09.006

[128] Upadhyay RP, Nayak C Homeopathy emerging as nanomedicine. Int J High Dil Res 2011;10:299–310

[129] Van Wassenhoven M, Goyens M, Capieaux E, et al. Nanoparticle characterisation of traditional homeopathically manufactured *Cuprum metallicum* and *Gelsemium sempervirens* medicines and controls. Homeopathy 2018;107:244–263 30144789 10.1055/s-0038-1666864

[130] Van Wassenhoven M, Goyens M, Henry M, et al. Nuclear magnetic resonance characterization of traditional homeopathically manufactured copper (*Cuprum metallicum*) and plant (*Gelsemium sempervirens*) medicines and controls. Homeopathy 2017;106:223–239 29157472 10.1016/j.homp.2017.08.001

[131] van Wijk R, Bosman S, van Wijk EPA Thermoluminescence in ultra-high dilution research. J Altern Complement Med 2006;12:437–443 16813507 10.1089/acm.2006.12.437

[132] Veith H. Dynamics of agitated fluids [In German]. Biologische Medizin 1976;5:123–125

[133] Walach H, van Asseldonk T, Bourkas P, et al. Electric measurement of ultra-high dilutions—A blinded controlled experiment. Br Homoeopath J 1998;87:3–12

[134] Weingärtner O. NMR-spectra of sulfur potencies [In German]. therapeutikon 1989;3:438–442

[135] Weingärtner O. Homeopathic potencies. Wish and reality in the search for therapeutically active components [In German]. Berlin: Springer Verlag, 1992.

[136] Witt C Attempt to measure an immaterial information [In German]. In: Albrecht H, Frühwald M, eds. Jahrbuch Karl und Veronica Carstens-Stiftung. Vol 2 Stuttgart: Hippokrates, 1995:153–165

[137] Witt C, Ludtke R, Weisshuhn TE, Willich SN High homeopathic potencies are different from potentized solvent when investigated with the REDEM technology. Forsch Komplementarmed Klass Naturheilkd 2005;12:6–13 15772457 10.1159/000082635

[138] Witt CM, Ludtke R, Weisshuhn TER, et al. The role of trace elements in homeopathic preparations and the influence of container material, storage duration, and potentisation. Forsch Komplementarmed Klass Naturheilkd 2006;13:15–21 10.1159/000090415 16582546

[139] Wolf U, Wolf M, Heusser P, et al. Homeopathic preparations of quartz, sulfur and copper sulfate assessed by UV-spectroscopy. Evid Based Complement Alternat Med 2011;2011:692798 19474239 10.1093/ecam/nep036 PMC3137246

[140] Wurmser L, Loch P Experimental research on homeopathic dilutions [In French]. In: L.H.I., ed. X. Kongress der Liga Homoeopathica Internationalis Budapest 1935. Budapest: o. V., 1935:359–373

[141] Zacharias CR. Implications of contaminants to scientific research in homoeopathy. Br Homeopath J 1995;84:3–5

[142] Zacharias CR. Contaminants in commercial homoeopathic medicines. Br Homeopath J 1995;84:71–74

[143] Zembrzuski W, Karbowska B Determination of Tl(I) ions in homeopathic drugs by differential pulse anodic stripping voltammetry. Indian J Pharm Educ Res 2017;51:620–625

[144] Hibou F. Could the study of cavitation luminescence be useful in high dilution research? Homeopathy 2017;106:181–190 28844291 10.1016/j.homp.2017.05.001

[145] Betti L, Trebbi G, Kokornaczyk MO, et al. Number of succussion strokes affects effectiveness of ultra-high-diluted arsenic on in vitro wheat germination and polycrystalline structures obtained by droplet evaporation method. Homeopathy: J Faculty Homeopathy 2017;106:47–54 10.1016/j.homp.2016.12.001 28325224

[146] Baumgartner S, Heusser P, Thurneysen A Methodological standards and problems in preclinical homoeopathic potency research. Forsch Komplementarmed Klass Naturheilkd 1998;5:27–32 10.1159/000021071 9761983

[147] Kovacs Z, Bazar G, Oshima M, et al. Water spectral pattern as holistic marker for water quality monitoring. Talanta 2016;147:598–608 26592651 10.1016/j.talanta.2015.10.024

[148] Pollack GH. The Fourth Phase of Water: Beyond Solid, Liquid, and Vapor. Seattle, WA: Ebner & Sons, 2013

[149] Konovalov AI, Ryzhkina IS Formation of nanoassociates as a key to understanding of physicochemical and biological properties of highly dilute aqueous solutions. Russ Chem Bull 2014;63:1–14

