# Supplementary material for: Physicochemical Investigations of Homeopathic Preparations: A Systematic Review and Bibliometric Analysis—Part 2
Source: J Altern Complement Med. 2019 Sep 12;25(9):890–901. doi: 10.1089/acm.2019.0064 (PMC6760181; doi:10.1089/acm.2019.0064)
Supplement: Supplemental data [file Supp_Table1.pdf]

Supplementary Data

Supplementary Table S1. Replications Using Analytical Methods

Experiment	*Silicea*	*Argentum metallicum*	*Publication*	*Average MIS*	*Potency level*	*Blinding*	*Randomization*	*Statistics*	*Independent production lots*	*Succussed controls*	*Differences reported*
Boyd1936-AnaM-1			BS	6.5	L	0	0	0	0	0	n
Boyd1936-AnaM-2			BS	6.5	L	0	0	0	0	0	n
Bonet-Maury1954			Pru	6.5	M	0	0	0	0	0	n
Lefebvre1978			PRu	6.5	L	0	0	0	0	1	n
Frisse1981-AnaM-1			T	6.5	L	0	0	0	0	0	n
Frisse1981-AnaM-2			T	6.5	L	0	0	0	0	0	n
Witt1995-AnaM			BS	8.5	L	0	0	0	0	0	n
Witt2006			PR	10	H	1	1	1	1	1	n
Baumgartner2009-AnaM			PR	9.5	M	1	1	0	0	1	n
Chikramane2010-AnaM		•	PR	6.5	M	0	0	0	0	0	y
Demangeat2010-AnaM_1	•	│	PR	10	M	1	1	0	1	1	y
Demangeat2010-AnaM-2	•	│	PR	10	M	0	1	0	1	1	n
Elia2010a-AnaM	│	│	PR	7.5	-	0	0	0	0	0	n
Upadhyay2011-AnaM	│	│	PR	8.5	M	0	0	0	0	1	n
Wolf2011-AnaM	•	│	PR	10	M	1	1	0	1	1	n
Chikramane2012		│	PR	8	M	0	0	0	0	0	y
Pillai2014		│	PR	6.5	H	0	0	0	1	0	n
Bell2015b-AnaM		•	PR	10	M	1	1	0	0	1	n
Chatterjee16_Ana			PR	5	M	0	0	0	0	0	n
Gayen18_Ana			PR	5	M	0	0	0	0	0	y
Holandino17_Ana			PR	9.5	L	0	0	1	0	1	n
Wassenhofen18_Ana			PR	9.5	M	0	1	1	0	1	n

Showing substances used in replication, individual experiments within a replication series are marked •. Showing in what type of publication it appeared (BS, book section; C, conference proceedings; nPR, nonpeer-reviewed; PR, peer-reviewed journal; PRu, journal peer-review unknown; T, thesis), average MIS score of the publications involved, potency levels used (L: low potencies, <12c, 24x; M: mixed; H: high, >12c, 24x), use of blinding, randomization, statistics, independent production lots, and succussed controls and finally whether differences were reported between homeopathic preparations and controls.

MIS, Manuscript Information Score.
