# Supplementary material for: Physicochemical Investigations of Homeopathic Preparations: A Systematic Review and Bibliometric Analysis—Part 2
Source: J Altern Complement Med. 2019 Sep 12;25(9):890–901. doi: 10.1089/acm.2019.0064 (PMC6760181; doi:10.1089/acm.2019.0064)
Supplement: Supplemental data [file Supp_Table2.pdf]

Supplementary Table S2. Replication Using Calorimetry

Experiment	*2.4D*	*Nat Mur*	*Indole*	*N-(phosphono methyl)-glycine*	*Arsenicum sulphuratum rubrum*	*Ars Alb*	*Arnica*	*Mag mur*	*Publication*	*Average MIS*	*Potency level*	*Blinding*	*Randomization*	*Statistics*	*Independent production lots*	*Succussed controls*	*Differences reported*
Dragan1992									PR	6.5	L	0	0	0	0	0	y
Anagnostatos 1992_1998-Cal									BS	6	M	0	0	0	0	1	y
Elia1999	•	•	•	•					PR	5	M	0	0	0	0	0	y
Elia2000	|	•	|	|					PR	8	M	0	0	0	0	0	y
Elia2004b-Cal	•	•	•	•					PR	7	M	0	0	0	0	0	y
Elia2004c-Cal	•				•				PR	7.5	M	0	0	0	0	0	y
Elia2005-Cal	•				•				PR	7.5	M	0	0	0	0	0	y
Elia2006a-Cal	•				|				PR	5	M	0	0	0	0	0	y
Elia2006b-Cal	•				•				PR	8	M	0	0	1	0	0	y
Elia2007b-Cal	•								PR	5.5	M	0	0	0	0	0	y
Belon2008-Cal	|					•	•	•	PR	6.5	M	0	0	0	0	0	y
Elia2008c	•					|	|	|	PR	6.5	M	0	0	0	0	0	y
Elia2010b-Cal	•					•	•	•	PR	6	M	0	0	0	0	0	y
Elia2013-Cal							•		PR	6.5	H	0	0	0	0	0	y
Elia2014a-Cal									PR	5.5	M	0	0	0	0	0	y
Holandino17_Cal									PR	9.5	L	0	0	1	0	1	n

MIS, Manuscript Information Score.
