# Supplementary material for: Physicochemical Investigations of Homeopathic Preparations: A Systematic Review and Bibliometric Analysis—Part 2
Source: J Altern Complement Med. 2019 Sep 12;25(9):890–901. doi: 10.1089/acm.2019.0064 (PMC6760181; doi:10.1089/acm.2019.0064)
Supplement: Supplemental data [file Supp_Table3.pdf]

Supplementary Table S3. Replications Using Chromatography

Experiment	*Arg Nit*	*Publication*	*Average MIS*	*Potency level*	*Blinding*	*Randomization*	*Statistics*	*Independent production lots*	*Succussed controls*	*Differences reported*
Maag1932	•	Pru	6	M	0	0	0	1	0	y
Maag1933	|	Pru	6	L	0	0	0	1	0	y
Kolisko1959	•	BS	5	M	0	0	0	1	0	y

MIS, Manuscript Information Score.
