# Supplementary material for: Physicochemical Investigations of Homeopathic Preparations: A Systematic Review and Bibliometric Analysis—Part 2
Source: J Altern Complement Med. 2019 Sep 12;25(9):890–901. doi: 10.1089/acm.2019.0064 (PMC6760181; doi:10.1089/acm.2019.0064)
Supplement: Supplemental data [file Supp_Table4.pdf]

Supplementary Table S4. Replications Using Electrochemistry

Experiment	*pH:Ars*	*pH:2.4D*	*pH:Arn*	*Publication*	*Average MIS*	*Potency level*	*Blinding*	*Randomization*	*Statistics*	*Independent production lots*	*Succussed controls*	*Differences reported*
Beier1953				T	10	M	0	0	0	0	0	y
Knauer1969-2	•			Pru	7	M	0	0	0	0	0	y
Heintz1971	|			nPR	10	M	0	0	0	0	1	y
Ciavatta2008	|			PR	9	M	0	0	0	0	0	y
Cacace2009-Echem	•	•	•	PR	8	M	0	0	0	0	0	y
Elia2009-Echem	•	•	•	PR	6	M	0	0	0	0	0	y
Sharma2012-Echem				PR	7	M	0	0	0	0	0	y
Bandyopadhyay17				PR	5	M	0	0	0	0	0	y
Zembruski17				PR	8	L	0	0	0	0	0	n

MIS, Manuscript Information Score.
