# Supplementary material for: Physicochemical Investigations of Homeopathic Preparations: A Systematic Review and Bibliometric Analysis—Part 2
Source: J Altern Complement Med. 2019 Sep 12;25(9):890–901. doi: 10.1089/acm.2019.0064 (PMC6760181; doi:10.1089/acm.2019.0064)
Supplement: Supplemental data [file Supp_Table5.pdf]

Supplementary Table S5. Replications Using Imaging Methods

Experiment	*GDV: Argentum met*	*Aurum met*	*Cuprum met*	*Ferr met*	*Gelse Semp*	*Nat Mur*	*Platinum met*	*Stannum met*	*Zinc met*	*Publication*	*Average MIS*	*Potency level*	*Blinding*	*Randomization*	*Statistics*	*Independent production lots*	*Succussed controls*	*Differences reported*
Mayrhofer1842ab	•	•	•	•			•	•	•	Pru	5.5	M	0	0	0	0	0	y
Jerman1999	│	│	│	│			│	│	│	PR	9	M	0	0	1	0	1	y
Bell2003	│	│	│	│		•	│	│	│	PR	9	H	1	1	1	0	0	y
Assumpcao2008-Img	│	│	│	│		•	│	│	│	PR	7.5	M	0	0	0	1	1	y
Chikramane2010-Img	•	•	•	│			•	•	•	PR	6.5	M	0	0	0	0	0	y
Upadhyay2011-Img	│	│	│	│					│	PR	8.5	M	0	0	0	0	1	y
Elia2014b-Img	│	│	│	│					│	PR	6	-	0	0	0	0	0	y
Bell2015a	│	│	│	│	•				│	PR	9.5	M	1	1	1	0	1	y
Bell2015b-Img	•	│	│	│	│				│	PR	10	M	1	1	1	0	1	y
Chatterjee16_Ima		│	│	•	│				│	PR	5	M	0	0	0	0	0	y
Paul16_Ima		│	│	•	│				│	PR	7	H	0	0	0	0	0	n
Temgire16		•	│		│				│	PR	7.5	M	0	0	0	0	0	n
Holandino17_Ima		│	│		│				•	PR	9.5	L	0	0	1	0	1	n
Kar17		•	│		│					PR	6	M	0	0	0	0	1	y
Wassenhofen18_Ima			•		•					PR	9.5	M	0	1	1	0	1	n
Gayen18_Ima										PR	5	M	0	0	0	0	0	n

GDV, gas discharge visualization; MIS, Manuscript Information Score.
