# Supplementary material for: Physicochemical Investigations of Homeopathic Preparations: A Systematic Review and Bibliometric Analysis—Part 2
Source: J Altern Complement Med. 2019 Sep 12;25(9):890–901. doi: 10.1089/acm.2019.0064 (PMC6760181; doi:10.1089/acm.2019.0064)
Supplement: Supplemental data [file Supp_Table6.pdf]

Supplementary Table S6. Replications Using Electrical Impedance Methods

Experiment	*2.4D*	*Aceticum acidum*	*Arn*	*Arg Nit*	*Arg Nit REDEM*	*Ars Alb*	*Ars sul*	*Aurum REDEM*	*Ferr Met*	*Mag Mur*	*Merc Dulcis*	*Nat mur*	*Plumb Nit*	*Sil*	*Uranyl nit*	*Publication*	*Average MIS*	*Potency level*	*Blinding*	*Randomization*	*Statistics*	*Independent production lots*	*Succussed controls*	*Differences reported*
Heintz1941-Imp		•									•	•			•	PR	6	M	0	0	0	0	0	y
Heintz1942-Imp		•									|	|			•	PR	5	M	0	0	0	0	0	n
Gay1951											|	|				C	6	M	0	0	0	0	0	y
Brucato1966											|	|				PR	5.5	M	0	1	0	1	0	y
Knauer1969-1				•							|	|	•			PRu	6.5	M	0	0	0	0	0	y
Knauer1970				•							•	|	•			PRu	6.5	M	0	0	0	1	0	y
Witt1995-Imp					•			•				|				B	8.5	H	1	1	0	0	1	y
Anagnostatos 1992_1998-Imp					|			|				|				B	6.5	M	0	0	0	0	1	y
Walach1998					|			|				|				PR	8	M	1	1	1	1	1	n
Elia2004a	•				|		•	|				|				PR	7	M	0	0	0	0	0	y
Elia2004b-Imp	•				|		|	|				•				PR	7	M	0	0	0	0	0	y
Elia2004c-Imp	•				|		•	|				|				PR	7.5	M	0	0	0	0	0	y
Süss2004-Imp-1	|				|		|	|				|				C	7.5	M	0	0	0	0	0	n
Süss2004-Imp-2	|				|		|	|				|				C	7.5	M	0	0	1	0	0	n
Chibici-Revneanu 2005-Imp	|				|		|	|				•				T	9.5	M	1	1	0	1	1	n
Elia2005-Imp	•				|		•	|				|				PR	7.5	M	0	0	0	0	0	y
Witt2000_2005	|				•		|	•				|				PR	10	H	1	1	1	1	1	y
Elia2006a-Imp	|		•				|			•		|		•		PR	5	M	0	0	0	0	0	y
Elia2006b-Imp	•		|				•			|		|		|		PR	8	M	0	0	0	0	0	y
Elia2007a	•		•				•			•		|		•		PR	6	M	0	0	0	0	0	y
Elia2007b-Imp	•		|							|		|		|		PR	5.5	M	0	0	0	0	0	y
Assumpcao2008-Imp	|		|							|		•		|		PR	7.5	M	0	0	0	1	1	y
Belon2008-Imp	|		•			•				•				|		PR	6.5	M	0	0	0	0	0	y
Elia2008a	|		•			|				•				•		PR	5.5	M	0	0	0	0	0	y
Elia2008b	|		•			|				|						PR	5.5	M	0	0	0	0	0	y
Holandino2008a	|		|			|				|						B	9	M	0	0	1	0	1	n
Holandino2008b	|		|			|				|						PR	8	M	0	0	0	0	1	n
Ramos2008	|		|			|				|						B	7.5	M	0	0	0	0	1	y
Cacace2009-Imp	•		•			•				•						PR	7.5	M	0	0	0	0	0	y
Elia2009-Imp	•		•			•				•						PR	6	M	0	0	0	0	0	y
Elia2010a-Imp	•		|			•				|						PR	7.5	-	0	0	0	0	0	y
Elia2010b-Imp	•		•			•				•						PR	6	M	0	0	0	0	0	y
Betti2011			|			•										PR	10	H	1	0	1	0	0	y
Elia2012			|													PR	5	M	0	0	0	0	0	y
Elia2013-Imp			•													PR	6.5	H	0	0	0	0	0	y
Mahata2013			|													PR	6	M	0	0	0	0	0	y
Elia2014a-Imp			|													PR	5.5	M	0	0	0	0	0	y
Elia2014b-Imp			•													PR	6	M	0	0	0	0	0	y
Chatterjee16_Imp									•							PR	5	M	1	0	0	1	1	y
Gayen18_Imp									|							PR	5	M	1	0	0	2	2	y
Paul16_Elec									•							PR	7	H	0	0	0	3	3	n

MIS, Manuscript Information Score.
