# Supplementary material for: Physicochemical Investigations of Homeopathic Preparations: A Systematic Review and Bibliometric Analysis—Part 2
Source: J Altern Complement Med. 2019 Sep 12;25(9):890–901. doi: 10.1089/acm.2019.0064 (PMC6760181; doi:10.1089/acm.2019.0064)
Supplement: Supplemental data [file Supp_Table7.pdf]

Supplementary Table S7. Replications Using Luminescence Techniques

Experiment	*Thermo: Lith Mur*	*Thermo: Mat mur*	*Delayed Lum: Arg Met*	*Delayed Lum: Canth*	*Publication*	*Average MIS*	*Potency level*	*Blinding*	*Randomization*	*Statistics*	*Independent production lots*	*Succussed controls*	*Differences reported*
Güldenstern2001					nPR	7	M	1	1	1	0	0	y
Lobyshev2001					C	5	M	0	0	0	0	0	y
Rey2003	•	•			PR	5	H	0	0	0	0	1	y
Lobyshev 2001_2005	|	|			PR	7	M	0	0	0	0	0	y
VanWijk2006	•	|			PR	10	H	1	1	1	0	1	y
Rey2007	•	•			PR	5	M	0	0	0	0	1	y
Sukul2007-Lum					PR	7	H	0	0	0	0	1	y
Bhattacharyya2008					PR	7	M	0	0	1	0	0	y
Lenger2008			•	•	PR	5.5	H	0	0	0	0	0	y
Sharma2012-Lum			|	|	PR	7	M	0	0	0	0	0	y
Lenger2014			•	•	PR	7.5	M	0	0	0	1	0	y

MIS, Manuscript Information Score.
