# Supplementary material for: Physicochemical Investigations of Homeopathic Preparations: A Systematic Review and Bibliometric Analysis—Part 2
Source: J Altern Complement Med. 2019 Sep 12;25(9):890–901. doi: 10.1089/acm.2019.0064 (PMC6760181; doi:10.1089/acm.2019.0064)
Supplement: Supplemental data [file Supp_Table8.pdf]

Supplementary Table S8. Replications Using NMR

Experiment	*Sulfur*	*Nux Vom*	*Silicea*	*Histamine*	*Publication*	*Average MIS*	*Potency level*	*Blinding*	*Randomization*	*Statistics*	*Independent production lots*	*Succussed controls*	*Result*
Smith1968					PR	5.5	M	1	1	0	0	0	y
Sacks1983					PRu	5.5	M	0	0	0	0	0	y
Lasne1985	•			•	nPR	6.5	M	0	0	1	0	1	y
Lasne1986	•			•	T	7	M	0	0	1	0	1	y
Weingärtner 1989_1990_1992	•			|	PRu	5.5	H	1	1	1	0	0	y
Demangeat1992	|		•	|	PR	9.5	M	1	1	1	1	1	y
Demangeat1997-1	|		•	|	C	9	M	1	1	1	1	1	y
Demangeat1997-2	|		•	•	C	9	M	1	1	1	1	1	y
Sukul2000	|	•	|	|	PR	7	H	0	0	1	0	1	y
Aabel2001-1	•	|	|	|	PR	7.5	M	0	0	1	0	0	n
Aabel2001-2	•	|	|	|	PR	7.5	M	0	0	1	0	0	n
Aabel2001-3	•	|	|	|	PR	7.5	H	0	0	1	0	0	n
Aabel2001-4	•	|	|	|	PR	7.5	H	0	0	1	0	0	n
Milgrom2001	|	|	|	|	PR	9.5	M	0	0	0	0	0	n
Sukul2001a	|	•	|	|	PR	8	H	0	0	1	0	1	y
Sukul2001c-NMR	|	•	|	|	PR	7	H	1	0	1	0	1	y
Anick2004-1	|		|	|	PR	9.5	H	0	1	0	0	0	n
Anick2004-2	|		|	|	PR	9.5	M	0	1	0	1	1	n
Demangeat2004	|		•	|	PR	10	M	1	1	1	1	1	y
Botha2008	|		|	|	PR	7.5	H	0	0	1	0	0	y
Baumgartner 2009-NMR-1	•		|	|	PR	9.5	M	1	1	1	0	1	n
Baumgartner 2009-NMR-2	•		|	|	PR	9.5	M	1	1	1	0	1	y
DeAlvarenga2009			|	|	PR	7	H	0	0	0	0	0	y
Demangeat2009			|	•	PR	10	M	1	1	1	1	1	y
Demangeat 2010-NMR-1			•	|	PR	10	M	1	1	1	1	1	y
Demangeat 2010-NMR-2			•	|	PR	10	M	0	1	1	1	1	y
Demangeat2013			•	|	PR	8.5	M	0	0	0	1	1	y
Demangeat2015-1				•	PR	8	M	0	0	1	1	1	y
Demangeat2015-2					PR	8	M	0	1	1	0	1	y
Wassenhofen17					PR	10	M	0	1	1	0	1	y

MIS, Manuscript Information Score; NMR, nuclear magnetic resonance.
