# Supplementary material for: Physicochemical Investigations of Homeopathic Preparations: A Systematic Review and Bibliometric Analysis—Part 2
Source: J Altern Complement Med. 2019 Sep 12;25(9):890–901. doi: 10.1089/acm.2019.0064 (PMC6760181; doi:10.1089/acm.2019.0064)
Supplement: Supplemental data [file Supp_Table9.pdf]

Supplementary Table S9. Replications Using Raman Spectroscopy

Experiment	*Bryonia*	*Nat mur*	*Publication*	*Average MIS*	*Potency level*	*Blinding*	*Randomization*	*Statistics*	*Independent production lots*	*Succussed controls*	*Differences reported*
Luu1974-Ram-1	•		T	8	L	0	0	0	0	0	y
Luu1974-Ram-2	•		T	8	L	0	0	0	0	0	y
Weingärtner 1992-Ram	•		BS	8.5	M	1	0	0	0	0	y
Rao2007		•	PR	8.5	M	1	0	0	0	0	y
Konar16		|	PR	7.5	H	0	0	0	0	1	n
Sarkar16a		•	PR	8.5	H	0	0	0	0	0	y
Sarkar16b_Ram			PR	6.5	M	0	0	0	0	1	y

MIS, Manuscript Information Score.
