# Supplementary material for: Physicochemical Investigations of Homeopathic Preparations: A Systematic Review and Bibliometric Analysis—Part 2
Source: J Altern Complement Med. 2019 Sep 12;25(9):890–901. doi: 10.1089/acm.2019.0064 (PMC6760181; doi:10.1089/acm.2019.0064)
Supplement: Supplemental data [file Supp_Table10.pdf]

Supplementary Table S10. Replications Using Spectroscopy Techniques

Experiment	*UV: Aconitum napellus*	*UV: Arnica Montana*	*UV: Apis*	*IR:Ferr Met*	*IR: Nat Mur*	*UV: Nat Mur*	*IR: Nux Vom*	*UV: Nux Vom*	*UV: Cuprum Sulf*	*UVVis: Glycerinum*	*UV: Sulfur*	*Publication*	*Average MIS*	*Potency level*	*Blinding*	*Randomization*	*Statistics*	*Independent production lots*	*Succussed controls*	*Differences reported*
Wurmser1935												C	6	M	0	0	0	0	0	y
Boyd1936-Spec								•				B	6.5	L	0	0	0	0	0	n
Heintz1941-Spec								|				PR	6	M	0	0	0	0	0	y
Heintz1942-Spec								|				PR	5	M	0	0	0	0	0	n
Taufiq1973	•							|				C	5	M	0	0	0	0	0	y
Veith1976	|							|				PR	5	M	0	0	0	0	0	y
Gautam1977	|							|				PRu	6.5	L	0	0	0	0	0	y
Zacharias1995a	|							|				PR	8.5	M	0	0	0	1	1	y
Zacharias1995b	|							|				PR	5.5	M	0	0	0	1	0	n
Sukul2001a-Spec-1	|							•				PR	8	H	0	0	0	0	0	y
Sukul2001a-Spec-2	|						•	|				PR	8	H	0	0	0	0	0	y
Sukul2001b	|						|	|				PR	5	H	0	0	0	0	0	y
Sukul2001c-Spec	|						|	•				PR	7	H	1	0	0	0	0	y
Gebhardt2002	|						|	|				MT	9	M	0	0	1	0	1	n
Chibici-Revneanu 2005-Spec	|					•	|	|				T	9.5	M	1	1	0	1	1	n
Sukul2005	|					|	•	|				PR	8.5	H	0	0	1	0	1	y
Rao2007-Spec-1	|					•	|	•				PR	8.5	M	1	0	0	0	1	y
Rao2007-Spec-2	|				•	|	•					PR	8.5	M	1	0	0	0	1	n
Sukul2007-Spec-1	|				|	|	|					PR	7	H	0	0	0	0	1	y
Marschollek2010	|				|	|	|		•		•	PR	9.5	M	1	1	1	0	1	y
Wolf2011-Spec	|				|	|	|		•		•	PR	10	M	1	1	1	1	1	y
Klein2013a	•				|	|	|		|		|	PR	8.5	M	1	0	1	0	0	y
Klein2013b	|				|	|	|		•		•	PR	9.5	M	1	1	1	0	1	y
Chakraborty2014	|				•	|	•				|	PR	5	M	0	0	0	0	0	y
Elia2014b-Spec-1	|	•				|					|	PR	6	M	0	0	0	0	0	y
Elia2014b-Spec-2	|	|				|					|	PR	6	H	0	0	0	0	0	y
Bell2015a-Spec	|	|				|					|	PR	10	M	1	1	1	0	1	y
Bell2015b-Spec	|	|				|					|	PR	10	M	1	1	1	0	1	y
Sarkar2015	|	|				|					|	PR	5.5	M	0	0	0	0	0	y
Cartwright2015	|	|				|				•	|	PR	8.5	H	0	0	1	0	0	y
Chatterjee16_Spec	|	|		•		|				|	|	PR	5	M	0	0	0	0	0	y
Klein16	•	|	•	|		|				|	•	PR	9	M	0	1	1	0	1	y
Paul16_Spec		|	|	•		|				|		PR	7	H	0	0	0	0	0	n
Pineros16		•	•			•				|		PR	8.5	H	0	0	1	0	0	y
Sarkar16b_Spec										|		PR	6.5	M	0	0	0	0	1	y
Cartwright17										•		PR	9.5	H	0	0	0	0	0	y
Cartwright18										•		PR	9.5	H	0	0	0	0	0	y
Gayen18_Spec												PR	5	M	0	0	0	0	0	y
Wassenhofen 18_Spec												PR	9.5	M	0	1	1	0	1	n

MIS, Manuscript Information Score; UV, ultraviolet.
