# Supplementary material for: Physicochemical Investigations of Homeopathic Preparations: A Systematic Review and Bibliometric Analysis—Part 2
Source: J Altern Complement Med. 2019 Sep 12;25(9):890–901. doi: 10.1089/acm.2019.0064 (PMC6760181; doi:10.1089/acm.2019.0064)
Supplement: Supplemental data [file Supp_Table11.pdf]

Supplementary Table S11. Surface Tension/Various Physical

Experiment	*UltraSon: Bryonia*	*Publication*	*Average MIS*	*Potency level*	*Blinding*	*Randomization*	*Statistics*	*Independent production lots*	*Succussed controls*	*Differences reported*
Silvio1990-Var-1	•	PR	6.5	M	0	0	0	0	0	n
Silvio1990-Var-2	•	PR	6.5	M	0	0	0	0	0	n
Silvio1990-Var-3		PR	6.5	L	0	0	0	0	0	n
Weingärtner 1992-Var		BS	8.5	M	1	0	0	1	0	n
Lenger2004		Pru	5.5	H	0	0	0	0	1	y
Bell2015a-Var		PR	9.5	M	1	1	1	0	1	y
Bell2015b-Var		PR	10	M	1	1	1	0	1	y
Nain2015		PR	8.5	M	0	0	0	0	1	y

MIS, Manuscript Information Score.
